# Generalized Robust Optimization using the Notion of Set-Valued Probability

**DOI:** 10.1007/s10957-025-02790-6

**Published:** 2025-08-29

**Authors:** Davide La Torre, Franklin Mendivil, Matteo Rocca

**Affiliations:** 1 SKEMA Business School, Université Côte d’Azur Sophia Antipolis Campus, Sophia Antipolis, France; 2 Department of Mathematics and Statistics, Acadia University, Wolfville, Nova Scotia Canada; 3 Department of Economics, Universitá degli Studi dell’Insubria, Varese, Italy

**Keywords:** Robustness, Set-Valued Probability Measure, Portfolio Optimization, Risk Measure, 49-XX, 60-XX

## Abstract

We propose a novel concept of robustness grounded in the framework of set-valued probabilities, offering a unified and versatile approach to tackling challenges associated with the statistical estimation of uncertain or unknown probabilities. By employing scalarization techniques for set-valued probabilities, we derive optimality conditions. Additionally, we establish generalized convexity properties and stability conditions, which further underpin the robustness of our approach. This comprehensive framework finds significant applications in areas such as financial portfolio management and risk measure theory, where it provides powerful tools for addressing uncertainty, optimizing decision-making, and ensuring resilience against variability in probabilistic models.

## Introduction

Robustness in optimization is fundamentally linked to the concepts of uncertainty and randomness. In many real-world scenarios, decision-makers face situations where parameters are not known with certainty and can vary unpredictably. Robust optimization seeks to create solutions that remain effective under a range of possible conditions, thereby mitigating the risks associated with this uncertainty. By accounting for potential variations and disturbances in input data, robust optimization aims to identify strategies that are less sensitive to fluctuations, ensuring that outcomes remain satisfactory even when faced with unpredictable circumstances.

In recent years, robust optimization has experienced significant growth in both research and practical applications. This paper explores the concept of generalized robustness by utilizing the idea of set-valued probability. The fundamental intuition of this approach is that the notion of set-valued probability encompasses all potential scenarios, including fluctuations and variations. When solving robust optimization models, we seek to identify solutions that maintain their effectiveness despite unforeseen changes or disruptions. We will establish scalarization results and optimality conditions in both convex and generalized convex contexts. A significant focus of this paper is on analyzing the difference between the robust problem and its nominal counterpart. This analysis is facilitated through appropriate scalarizations which depend on the configuration of the set-valued probability. Additionally, we conduct a sensitivity analysis of robust solutions in relation to variations in this set-valued probability. The Monge-Kantorovich distance between set-valued probabilities will serve as a key metric for quantifying the loss associated with the transition from the nominal to the robust problem.

The research presented in this paper introduces methodologies that can be applied to a variety of financial challenges, particularly within the realms of robust portfolio optimization and risk measure theory. Robust portfolio optimization plays a pivotal role in ensuring portfolio stability amidst market fluctuations and enhancing resilience against unforeseen changes. Additionally, the integration of these techniques into risk measure theory provides a powerful framework for quantifying and managing financial risks. These methodologies are highly adaptable and can be customized to address a broad spectrum of portfolio optimization and risk management problems. Their versatility underscores their substantial relevance and potential impact, offering finance, investment, and risk management professionals valuable tools for improving portfolio decision-making and mitigating risks effectively [17–19].

The structure of the paper is organized as follows. In Sect. 2, we revisit the framework of robust optimization problems in the context of classical probability measures. This section emphasizes the traditional approaches to robust optimization and sets the stage for a deeper exploration of how these concepts can be adapted and enhanced. In Sect. 3, we explore key properties and definitions within the realm of set-valued analysis, which serve as a foundational framework for the subsequent discussions. This section also introduces the concept of minimization with respect to set-inclusion ordering, highlighting its significance in understanding how sets can be compared and optimized. Moving forward, Sect. 4 analyzes the notion and properties of set-valued probabilities. Here, we examine how these probabilities can be utilized to capture uncertainty, allowing for a better representation of complex scenarios. Section 5 presents the robust optimization model specifically designed for set-valued probabilities. We discuss the implications of this model, demonstrating its ability to maintain effectiveness and stability in the face of uncertainty. The theoretical foundations are further developed in Sect. 6, where we focus on significant results derived from scalarization techniques. This section aims to bridge the gap between theoretical constructs and practical applications, providing a clear understanding of how these results can be leveraged. Section 7 illustrates the application of these concepts to robust portfolio theory, showcasing how the methodologies discussed can enhance decision-making in investment strategies. This real-world application underscores the importance of robust optimization in achieving resilience in financial portfolios. Finally, Sect. 8 summarizes the key findings of the paper, reflecting on their implications for future research and practice. This concluding section aims to reinforce the relevance of robust optimization and set-valued probabilities in addressing contemporary challenges in decision-making.

## Preliminaries on Robust Optimization

Consider a sample space $(\varOmega , \mathcal {B}, \mu )$, where $\varOmega$ represents the space of events, $\mathcal {B}$ is the Borel $\sigma$-algebra on $\varOmega$, and $\mu$ denotes a probability measure. Let $\omega \in \varOmega$ be a specific event in the space $\varOmega$, and let *X* denote a subset of ${\mathbb {R}}^n$. We study a stochastic problem of the following form:

1 $$\begin{aligned} \textrm{min}_{x \in X} \xi (x, \omega ), \end{aligned}$$

where $\xi : X\times \varOmega \rightarrow {\mathbb {R}}$ is a scalar function. Associated with every function $\xi$ there is a vector

$$\begin{aligned} ({{\mathcal {F}}}^{(1)}(\xi (x, \omega ), \mu ), \ldots , {{\mathcal {F}}}^{(r)}(\xi (x, \omega ), \mu )) \end{aligned}$$

consisting of functionals ${{\mathcal {F}}}^{(s)}(\xi (x, \omega ), \mu )$ of $\xi (x, \omega )$ which may represent the expectation, variance, a quantile, a risk measure, or other summary statistics of the random variable $\xi (x, \omega )$. In this manner, we link problem (1) to a deterministic multiobjective optimization problem (see [5–7, 23]) expressed as:

$$\begin{aligned} \textrm{min}_{x \in X} (z^{(1)}(x, \mu ), \ldots , z^{(r)}(x, \mu )) \end{aligned}$$

where $$z^{(s)}(x, \mu )={{\mathcal {F}}}^{(s)}(\xi (x, \omega ), \mu )$$.

In the previous model, we assume that the probability distribution $\mu$ on $\varOmega$ is known. However, in real-world situations, our understanding of the statistical properties of the model parameters is often incomplete, and the corresponding probability distributions are never fully specified ( [9, 10]). Specifically, the probability distribution that captures the uncertainty of the model parameters is known only ambiguously. A common method to address this ambiguity, from a statistical standpoint, is to estimate the probability distribution using empirical samples. As a result, the decision-making process can be based on this estimated distribution. However, this statistical approach may lead to imprecise conclusions, particularly due to the influence of outliers or errors in the sampling process [11].

Another method for managing ambiguity involves the assumption that the underlying probability distribution is unknown and lies within an ambiguity set of distributions. In this case, ambiguity in probability distributions is addressed using a worst-case (min-max) strategy. This methodology is usually referred to as Distributionally Robust Optimization. Let us assume, for simplicity, that ${{\mathcal {F}}}^{(1)}(\xi _1(x, \omega ), \mu ) ={\mathbb {E}}_{\mu }(\xi (x, \cdot ))=\int _{\varOmega } \xi (x,\omega ) d\mu (\omega )$. If we suppose that a family ${{\mathcal {A}}}$ of probability measures is provided ($\mathcal {A}$ models the set of all possible distributions that are relevant for the considered problem), then one may hedge against the worst expected value resulting from the distributions in the set $\mathcal {A}$ by solving the following distributionally robust optimization problem ( [8]):

2 $$\begin{aligned} \textrm{min}_{x \in X}\ \textrm{max}_{\mu \in {{\mathcal {A}}}} \ \ {\mathbb {E}}_{\mu }(\xi (x,\cdot )). \end{aligned}$$

The following section focuses on extending this formulation using the concept of set-valued probability. The core idea of this new notion of robustness is to introduce a new object - a set-valued probability measure - that encompasses all possible probability measures within $\mathcal {A}$.

## Set-Valued Functions

We now provide some foundational definitions and concepts from the theory of convex sets and set-valued functions. For further details, refer to [2, 4, 14]. Throughout, we denote by ${\mathcal {K}}$ the collection of all nonempty, compact, and convex subsets of $\mathbb {R}^d$. We add elements of ${\mathcal {K}}$ or multiply by a scalar ($\lambda \in {\mathbb {R}}$) using $A + B := \{ a + b : a \in A, b \in B \} \text{ and } \lambda A = \{ \lambda a : a \in A \}$. For $A \in {\mathcal {K}}$, we say that *A is nonnegative* ($A \ge 0$) if $0 \in A$. The *support function* $spt(\cdot , A):{\mathbb {R}}^d \rightarrow {\mathbb {R}}$ for $A \in {\mathcal {K}}$ and $p \in \mathbb {R}^d$ is given by $spt(p,A) = \sup \{ p \cdot a : a \in A \}$. This function completely defines *A* since

3 $$\begin{aligned} A = \bigcap _{\Vert p \Vert = 1 } \{ x : x\cdot p \le spt(p,A) \}. \end{aligned}$$

Notice $A \ge 0$ iff $spt(p,A) \ge 0$ for all *p*. Furthermore, $A\subseteq B$ if and only if $spt(p,A)\le spt(p,B)$ for all $\Vert p\Vert =1$. In addition, for any $\lambda \ge 0$ and $A,B \in {\mathcal {K}}$, we have that $spt(p, \lambda A + B) = \lambda spt(p, A) + spt(p,B), \ spt(p,-B) = spt(-p,B)$. Normally, however, $spt(p,-A) \ne -spt(p,A)$. For any $A \in {\mathcal {K}}$, we can also define the *norm* of *A* as $\Vert A \Vert := \sup \{ \Vert x \Vert : x \in A \} = \sup _{\Vert p \Vert = 1 } spt(p,A)$. This definition satisfies all of the classical properties of a norm. The Hausdorff distance between $A,B \in {\mathcal {K}}$ can also be given in terms of the support function:

$$ d_H(A,B) = \sup _{\Vert p \Vert = 1} |spt(p,A) - spt(p,B)|. $$

This can be used to easily show that both addition and scalar multiplication on ${\mathcal {K}}$ are continuous in the Hausdorff distance.

For any set $A \subset {\mathbb {R}}^d$, we use $\text {conv}(A)$ to denote the smallest convex set which contains *A*. Note that if *A* is bounded then $\text {conv}(A) \in {\mathcal {K}}$.

A set $A \subset {\mathbb {R}}^d$ is *balanced* if $\lambda A \subseteq A$ for all $|\lambda | \le 1$. For us a *unit ball* in ${\mathbb {R}}^d$ is any balanced set ${\mathbb {B}}\in {\mathcal {K}}$ with $0 \in int ({\mathbb {B}}$). Any such unit ball defines a norm on ${\mathbb {R}}^d$ via the Minkowski functional

$$ \Vert x \Vert _{\mathbb {B}}= \sup \{ \lambda \ge 0 : \lambda x \in {\mathbb {B}}\}. $$

Given a unit ball ${\mathbb {B}}$, the *dual sphere* is defined as

$$ {{\mathbb {S}}}^* = \{ y : \sup \{ y \cdot x : x \in {\mathbb {B}}\} = 1 \} \subset {\mathbb {R}}^d $$

and is also a nonempty compact set. Notice that since ${\mathbb {B}}$ is compact, for each $y \in {{\mathbb {S}}}^*$, there is some $x \in {\mathbb {B}}$ with $y \cdot x = 1$. For more details see [22].

A *set-valued function* or *multifunction* taking compact and convex values is a map from ${\mathbb {R}}^n$ to ${\mathcal {K}}$. For a given set-valued function $f:{\mathbb {R}}^n \rightarrow {\mathcal {K}}$ and measure $\mu$, we can define the integral of *f* with respect to $\mu$ as an element of ${\mathcal {K}}$ via support functions using the property (see [4])

$$ spt\left( q, \int _{{\mathbb {R}}^n} f(x) \ d\mu (x) \right) = \int _{{\mathbb {R}}^n} spt( q, f(x)) \ d\mu (x), $$

which defines the set as in (3). For more results on set-valued analysis see [4].

Given a compact subset $\varTheta$ of ${\mathbb {R}}^n$ and a set-valued function $f:\varTheta \subseteq {\mathbb {R}}^n\rightarrow {\mathcal {K}}$, consider the optimization problem

$$\begin{aligned} \max _{x\in \varTheta } f(x). \end{aligned}$$

A set-valued function $f:{\mathbb {R}}\rightarrow {\mathcal {K}}$ is *non decreasing* if $f(x) \subseteq f(y)$ whenever $x \le y$. A set-valued function $f:{\mathbb {R}}^n\rightarrow {\mathcal {K}}$ is *convex* if

4 $$\begin{aligned} f(t x + (1-t) y ) \subseteq t f(x) + (1-t) f(y) \end{aligned}$$

for $$x,y\in {\mathbb {R}}^n$$, $$t\in [0,1]$$.

Using the support function, we have that *f* is convex if and only if the function $spt(p,f(x))$ is convex for all $\Vert p \Vert =1$. *f* is said to be quasi-convex if the following property holds: for any $x,y\in {\mathbb {R}}^n$, with $f(x)\subseteq f(y)$, we have that

5 $$\begin{aligned} f(tx+(1-t)y)\subseteq f(y) \end{aligned}$$

for any $t\in [0,1]$. Finally we say that *f* is *convex by arcs* if

6 $$\begin{aligned} f(\gamma _{x,y}(t)) \subseteq t f(x) + (1-t) f(y) \end{aligned}$$

for any continuous function $\gamma _{x,y}(t):[0,1]\rightarrow {\mathbb {R}}^n$ such that $\gamma _{x,y}(0)=x$ and $\gamma _{x,y}(1)=y$.

## Set-Valued Probabilities

We give a brief overview of those aspects of set-valued measures and set-valued probabilities that we will need; for more information and proofs see [1, 2, 4, 12, 24, 25]. The notion of set-valued probability extends the classical notion of probability. Within this context we suppose that the probability of a certain event is no longer a positive number but a set: this definition can be very useful to model situations in which there is total ignorance and absolutely no information about the system or subject under study. This notion also extends the notion of imprecise probability [26–28] to the case of probabilities taking convex and compact-valued images. Within the imprecise probability formulation a single probability specification $\phi$ is replaced with an interval specification by means of lower and upper probabilities, namely $\phi _{-}$ and $\phi _{+}$, and for a given event *A* we have that $\phi (A)$ is replaced by the positive interval $[\phi _{-}(A),\phi _{+}(A)]\subset [0,1]$. The definition of imprecise probability can be recast in the framework of set-valued probability theory by assuming that the probability of a given event *A* is the interval $[-\phi _{-}(A),\phi _{+}(A)]\subset [-1,1]$.

Starting with a given set $\varOmega$ and a $\sigma$-algebra ${\mathcal {A}}$ on $\varOmega$, a *probability measure* on $(\varOmega ,{\mathcal {A}})$ with values in [0, 1] is a function $\mu : {\mathcal {A}}\rightarrow [0,1]$ such that $\mu (\emptyset ) = 0$, $\mu (\varOmega )=1$, and

$$ \mu \left( \bigcup _i A_i \right) = \sum _i \mu (A_i) $$

for any pairwise-disjoint sequence $A_i \in {\mathcal {A}}$. In a similar way, a *vector-valued probability measure* on $(\varOmega ,{\mathcal {A}})$ is a function ${\boldsymbol{\mu }}: {\mathcal {A}}\rightarrow [0,1]^d$, with ${\boldsymbol{\mu }}(\emptyset ) = (0,\ldots ,0)\in {\mathbb {R}}^d$, ${\boldsymbol{\mu }}(\varOmega )=(1,\ldots ,1)\in {\mathbb {R}}^d$, and

$$ {\boldsymbol{\mu }}\left( \bigcup _i A_i \right) = \sum _i {\boldsymbol{\mu }}(A_i) $$

Here additivity is satisfied componentwise. Note that with this definition a vector-valued probability measure is simply a vector of probability measures.

The next extension is that of a *set-valued measure* or *multimeasure* on $(\varOmega ,{\mathcal {A}})$ with values in ${\mathcal {K}}$. This is a function $\varPhi : {\mathcal {A}}\rightarrow {\mathcal {K}}$ such that $\varPhi (\emptyset ) = \{ 0 \}$ and

7 $$\begin{aligned} \varPhi \left( \bigcup _i A_i \right) = \sum _i \varPhi (A_i) \end{aligned}$$

for any sequence of disjoint sets $A_i \in {\mathcal {A}}$. The right side of (7) is the infinite Minkowski sum defined as

$$ \sum _i K_i = \left\{ \sum _i k_i : k_i \in K_i, \sum _i |k_i| < \infty \right\} . $$

Equivalently, we could require Hausdorff convergence for the sum in (7). We mention that our assumption that $\varPhi (A)$ is convex is not much of a restriction since this is always true for any bounded, non-atomic set-valued measure (see [1, 12]).

We will say that a multimeasure $\varPhi$ is *nonnegative*, and we write $\varPhi (A) \ge 0$, if $0 \in \varPhi (A)$ for all *A*. Nonnegative multimeasures are monotone: if $A \subseteq B$ then

$$ \varPhi (A) = \{ 0 \} + \varPhi (A) \subseteq \varPhi (B \setminus A) + \varPhi (A) = \varPhi (B). $$

This makes nonnegative multimeasures a nice generalization of (nonnegative) scalar measures which are also monotone.

If $\varPhi$ is a multimeasure and $p \in {\mathbb {R}}^d$ then the *scalarization* $\varPhi ^p$ defined by

$$\begin{aligned} \varPhi ^p(A) = spt(p,\varPhi (A)) \end{aligned}$$

is a signed measure on $\varOmega$ in general and a (positive) measure if $\varPhi$ is nonnegative. This is because $0 \in \varPhi (A)$ means $0 \le spt(p,\varPhi (A)) = \varPhi ^p(A)$.

### Definition 4.1

Let ${\mathbb {B}}\in {\mathcal {K}}$ be a unit ball. A ${\mathbb {B}}$ *set-valued probability* or *probability multimeasure* (or *pmm*) on $(\varOmega ,{\mathcal {A}})$ is a nonnegative multimeasure $\varPhi$ with $\varPhi (\varOmega ) = {\mathbb {B}}$.

Our assumptions on a pmm $\varPhi$ mean that $\varPhi ^p$ is a probability measure on $\varOmega$ for each $p \in {{\mathbb {S}}}$. To see this, just notice that $\varPhi ^p(A)$ is nonnegative, monotone in *A*, and $\varPhi ^p(\varOmega ) = spt(p,{\mathbb {B}}) = 1$. Note that $\varPhi ^p$ and $\varPhi ^q$ are not “independent” but may be related in a complex manner. The primary constraint is that for any measurable $A \subset \varOmega$, the mapping $p \mapsto \varPhi ^p(A)$ is convex.

One simple and useful way to construct a multimeasure is by integrating a *set-valued density function* *f* with respect to a positive scalar measure $\mu$:

8 $$\begin{aligned} \varPhi (A) = \int _A f(x) \ d\mu (x). \end{aligned}$$

This integral can be defined in several different ways (see [4]). If the set-valued function *f* is nonnegative (that is, $0 \in f(x)$ for all *x*), then the resulting multimeasure will also be nonnegative. In addition, if $0 \le f(x) \le g(x)$ and $\varPhi$ is a positive multimeasure, then

$$ \int f(x) \ d\varPhi (x) \subseteq \int g(x) \ d\varPhi (x), $$

for this to be true the convexity of the values of $\varPhi$ is crucial.

### Example 4.1

Let $\mu = (\mu _1, \mu _2, \dots , \mu _d)$ be a vector-valued probability measure, where each $\mu _i$ is a probability measure on a measurable space $(\varOmega , \mathcal {F})$. We define a corresponding set-valued measure $\varPhi$ with polyhedral values as follows:

$$ \varPhi (A) = \text {co} \left\{ 0, \pm \mu _i(A) e_i \mid i = 1, \dots , d\right\} , $$

for every $A \in \mathcal {F}$, where:

- $\mu _i(A)$ is the value of the probability measure $\mu _i$ for the set $A$,
- $e_i$ is the $i$-th standard basis vector in ${\mathbb {R}}^d$,

First, let us show an example in 2D. Consider the set-valued probability defined by:

$$ \phi (A) = \text {conv}\{\mu _1(A)e_1, \mu _2(A)e_2, -\mu _1(A)e_1, -\mu _2(A)e_2\}. $$

For the probabilities:

$$ \mu _1(A) = 0.6, \quad \mu _2(A) = 0.4, $$

the vertices of $\phi (A)$ are:

$$ (0.6, 0), \, (0, 0.4), \, (-0.6, 0), \, (0, -0.4). $$

**Fig. 1:**
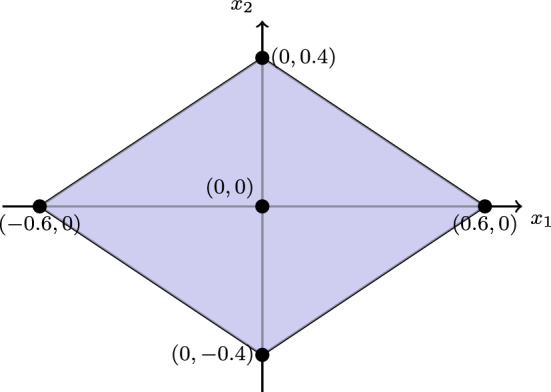
2D set-valued probability

See Fig. 1 for a geometric illustration of $\phi (A)$.

Let us now consider the following 3D example. We illustrate the integration of $g(x) = x$ with respect to the more involved pmm $\varPhi$ defined on the space $\varOmega = [-1,1]$ and using a density as in (8):

$$ \varPhi (A) = \int _A \text {conv}\{0, 2x e_1, 4 x^3 e_2, 6 x^5 e_3\} \ dx. $$

Since $$g(x) < 0$$ on $$[-1,0)$$ and $$g(x) > 0$$ on (0, 1], we need to split the integral into the two parts

$$\begin{aligned} \int _\varOmega g(x) \ d\varPhi (x)&= -\int _{-1}^0 -x d\varPhi (x) + \int _0^1 x \ d\varPhi (x)\\&= -\text {conv}\left\{ 0,\frac{-2 e_1}{3}, \frac{-4 e_2}{5}, \frac{-6 e_3}{7}\right\} + \text {conv}\left\{ 0,\frac{2 e_1}{3}, \frac{4 e_2}{5}, \frac{6 e_3}{7}\right\} \\&= \text {conv}\left\{ 0,\frac{4 e_1}{3}, \frac{8 e_2}{5}, \frac{12 e_3}{7}\right\} . \end{aligned}$$

Notice that the Minkowski sum of two polyhedra is the convex hull of the points we get when we take all pairwise sums of one extreme point from the first set and another from the second.

**Fig. 2:**
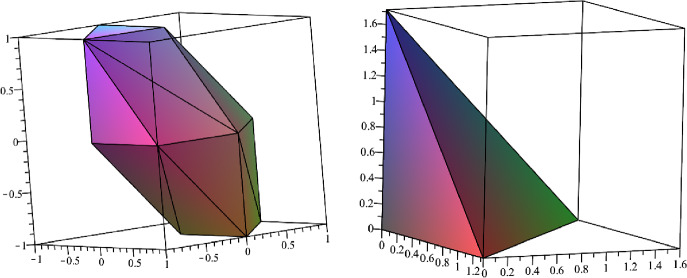
Unit ball and integral for Example 4.1

We point out that $\varPhi$ is indeed a pmm as

$$ \varPhi (\varOmega ) = \text {conv}\{0,-e_1,-e_2,-e_3\} + \text {conv}\{0,e_1,e_2,e_3\}, $$

which is a symmetric (balanced) convex set with 0 in its interior (see Fig. 2).

A *random variable* on $(\varOmega ,{\mathcal {A}})$ is simply a Borel measurable function $X:\varOmega \rightarrow {\mathbb {R}}$. The expectation of *X* with respect to a pmm $\varPhi$ is defined in the usual way as

$$\begin{aligned} {\mathbb {E}}_\varPhi (X) := \int _\varOmega X(\omega ) \ d\varPhi (\omega ). \end{aligned}$$

The integral can be constructed using support functions (that is, using the $\varPhi ^p$) and each part of the decomposition $X = X^+ - X^-$ separately (since support functions work best with nonnegative scalars); see [13] for another approach. Since $0 \in \varPhi (A)$ for each *A*, it is easy to see that $0 \in {\mathbb {E}}_{\varPhi }(X)$ as well.

Classical results like the strong law of Large Numbers, the Glivenko-Cantelli and the Central Limit Theorems can be extended to pmm (see [18] for more on this). We will denote by ${\textbf{M}}$ the space of all probability multimeasures defined on $\varOmega$. The Monge-Kantorovich distance can be extended to probability multimeasures by

$$ d_{{\textbf{M}}}(\varPhi _1,\varPhi _2) = \sup _{p \in {{\mathbb {S}}}^*} d_M(\varPhi _1^p,\varPhi _2^p), $$

where $d_M$ is the classical Monge-Kantorivich distance between probability measures (see [16, 21]).

## Generalized $\varPhi$-Robustness

In this section, we introduce a novel concept of generalized robustness, utilizing the framework of set-valued probability. This new notion is designed to encompass a comprehensive range of classical probability measures, allowing for a more flexible and inclusive analysis of uncertainty in decision-making processes. By leveraging set-valued probability, we aim to aggregate insights from a variety of classical probability distributions, thereby presenting a unified approach to modeling uncertainty.

The essence of set-valued probability lies in its ability to represent uncertainty not as a single probability measure but as a collection of potential measures. This aggregation allows for a richer representation of the uncertainty landscape, accommodating the inherent variability and ambiguity that often characterize real-world scenarios. By considering a set of classical probability measures, our model captures a broader spectrum of possible outcomes, effectively addressing the limitations of relying on a singular probability distribution.

As a result, the framework we propose is inherently set-valued by construction. Decision-makers can now evaluate strategies based on a comprehensive understanding of the uncertainties involved, rather than being constrained by a fixed probabilistic framework. This flexibility is particularly valuable in contexts where the underlying probability distributions are difficult to ascertain or subject to significant variability. Without losing any generality, in the following we assume that $\xi (x,\omega )\ge 0$ for any $x\in X$ and $\omega \in \varOmega$.

### Definition 5.1

Given a scalar or vector probability measure $\mu$ and a set-valued probability $\varPhi$ such that $\mu (A)\in \varPhi (A)$, the generalized $\varPhi$-robust model associated with the nominal problem

$$\begin{aligned} \textrm{min}_{x \in X}\ {\mathbb {E}}_{\mu }(\xi (x,\cdot )):= \int _\varOmega \xi (x,\omega ) d\mu (\omega ) \end{aligned}$$

is the following optimization problem

9 $$\begin{aligned} \textrm{min}_{x \in X}\ {\mathbb {E}}_{\varPhi }(\xi (x,\cdot )):= \int _\varOmega \xi (x,\omega ) d\varPhi (\omega ). \end{aligned}$$

The robust optimization problem is set-valued by construction, as the function $x\rightarrow {\mathbb {E}}_{\varPhi }(\phi (x,\cdot ))$ is a set-valued function. Let us also notice that $\mu$ and $\varPhi$ must take values in the same space of outcomes (so, for instance, if $\mu$ is a classical probability measure then $\varPhi$ is an interval-valued probability).

### Definition 5.2

A point $x^*\in X$ is a *global ideal minimizer* for the problem Eq. (9) if ${\mathbb {E}}_{\varPhi }(\xi (x^*,\cdot ))\subseteq {\mathbb {E}}_{\varPhi }(\xi (x,\cdot ))$ for any $x\in X$.

### Definition 5.3

A point $x^*\in X$ is a *global strong minimizer* for the problem Eq. (9) if there exists no $x\in X$, $x\not = x^*$, such that ${\mathbb {E}}_{\varPhi }(\xi (x,\cdot ))\subseteq {\mathbb {E}}_{\varPhi }(\xi (x^*,\cdot ))$.

### Definition 5.4

A point $x^*\in X$ is a *global minimizer* for the problem Eq. (9) if there exists no $x\in X$ such that ${\mathbb {E}}_{\varPhi }(\xi (x,\cdot ))\subset \textrm{int} \, {\mathbb {E}}_{\varPhi }(\xi (x^*,\cdot ))$.

It is clear that the following implications hold:

$$ \text {Ideal Minimizer} \implies \text {Strong Minimizer} \implies \text {Minimizer} $$

An interesting point to be discussed is the relationship between classical probability measures and the notion of set-valued measure. Suppose that $\mu$ is a classical probability measure that satisfies the property $\mu (A)\in \varPhi (A)$ for any set $A\in \mathcal {B}$. Now suppose that $\varPhi$ takes interval values and $\varPhi (\varOmega )=[0,1]$. Associated with $\mu$, let us define a set-valued probability measure

$$ \varPhi _\mu (A) = [0,\mu (A)]. $$

Since $0 \in \varPhi (A)$ for all *A*, the condition $\mu (A) \in \varPhi (A)$ implies that $\varPhi _\mu (A) = [0,\mu (A)] \subseteq \varPhi (A)$ (as $\varPhi (A)$ is a convex and contains both 0 and $\mu (A)$) and thus

10 $$\begin{aligned} {\mathbb {E}}_{\varPhi _\mu }(\xi (x,\cdot )) = [0,{\mathbb {E}}_{\mu }(\xi (x,\cdot ))] \subseteq {\mathbb {E}}_{\varPhi }(\xi (x,\cdot )) \end{aligned}$$

and, therefore,

11 $$\begin{aligned} \min _{x \in X} {\mathbb {E}}_{\varPhi _\mu }(\xi (x,\cdot )) \subseteq \min _{x \in X}\ {\mathbb {E}}_{\varPhi }(\xi (x,\cdot )). \end{aligned}$$

This says that the minimizer for $\varPhi _\mu$ is at least as good as that for $\varPhi$. This makes sense since $\mu$ being a selection of $\varPhi$ implies that the model which uses $\mu$ is less “uncertain” than the one which uses $\varPhi$; reducing uncertainty should not result in a worse optimal situation! By doing so, it provides a comprehensive framework that captures the entire range of probability measures represented by $\varPhi$, thereby offering a more robust and flexible approach to modeling uncertainty.

### The Notion of $\varPhi _{average}$-Robustness

The aim of this subsection is to introduce a notion of a $\varPhi _{average}$ robust optimization problem associated with the problem:

12 $$\begin{aligned} \textrm{min}_{x \in X}\ {\mathbb {E}}_{\varPhi }(\xi (x,\cdot )):= \int _\varOmega \xi (x,\omega ) d\varPhi (\omega ). \end{aligned}$$

and to explore links with $\varPhi$-robustness.The definition of $\varPhi _{average}$ robustness relies on the notion of the scalarized probability measures $\varPhi ^p$ of $\varPhi$.

We can now define a probability measure $\varPhi _{average}$ by integrating $\varPhi ^p$ as follows:

13 $$\begin{aligned} \varPhi _{average}(A) = \int _{{{\mathbb {S}}}^*} \varPhi ^p(A) dp. \end{aligned}$$

#### Definition 5.5

The generalized $\varPhi _{average}$-robust model is defined as

$$\begin{aligned} \textrm{min}_{x \in X}\ {\mathbb {E}}_{\varPhi _{average}}(\xi (x,\cdot )):= \int _\varOmega \xi (x,\omega )d\varPhi _{average}(\omega ). \end{aligned}$$

The notion of $\varPhi _{average}$ robustness aims to evaluate the overall performance or robustness of a solution by integrating (or averaging) its performance across a range of scalarizations. This ensures that the solution is not overly sensitive to any specific scalarization.

The following theorem analyzes the relationship between the notion of $\varPhi$ and $\varPhi _{average}$ robustness.

#### Theorem 5.1

If $x^*\in X$ is a global ideal minimizer of the $\varPhi$-robust model then it also solves the $\varPhi _{average}$-robust formulation. Furthermore, if $x^*$ is a strict global minimizer of the $\varPhi _{average}$ model, then it is a global strong minimizer of the $\varPhi$-robust formulation.

#### Proof

If $x^*$ is an ideal minimizer, then we have that for any $x\in X$

$$ {\mathbb {E}}_{\varPhi }(\xi (x^*,\cdot ))\subseteq {\mathbb {E}}_{\varPhi }(\xi (x,\cdot )). $$

By taking the support function along any direction $p\in {{\mathbb {S}}}^*$, we have

$$ {\mathbb {E}}_{\varPhi ^p}(\xi (x^*,\cdot )) = spt({\mathbb {E}}_{\varPhi }(\xi (x^*,\cdot )),p)\le spt({\mathbb {E}}_{\varPhi }(\xi (x,\cdot )),p) = {\mathbb {E}}_{\varPhi ^p}(\xi (x,\cdot )), $$

and so we have that

$$ {\mathbb {E}}_{\varPhi _{average}}(\xi (x^*,\cdot )) =\int _\varOmega \xi (x^*,\omega ) d\varPhi _{average}(\omega ) = \int _{{{\mathbb {S}}}^*} \int _\varOmega \xi (x^*,\omega ) d\varPhi ^p(\omega ) dp= $$

$$ \int _{{{\mathbb {S}}}^*} {\mathbb {E}}_{\varPhi ^p}(\xi (x^*,\cdot )) dp \le \int _{{{\mathbb {S}}}^*} {\mathbb {E}}_{\varPhi ^p}(\xi (x,\cdot )) dp = {\mathbb {E}}_{\varPhi _{average}}(\xi (x,\cdot )). $$

Thus $x^*$ is an ideal minimizer for the $\varPhi _{average}$ robust problem.

Now suppose that $x^*$ is a strict minimizer for the $\varPhi _{average}$ model. Then we have that

$$ {\mathbb {E}}_{\varPhi _{average}}(\xi (x,\cdot )) > {\mathbb {E}}_{\varPhi _{average}}(\xi (x^*,\cdot )). $$

This implies that for any $x\in X$ there exists a $p \in {{\mathbb {S}}}^*$ such that

$$ {\mathbb {E}}_{\varPhi ^{p}}(\xi (x,\cdot ))> {\mathbb {E}}_{\varPhi ^{p}}(\xi (x^*,\cdot )) $$

and this implies that

$$ {\mathbb {E}}_{\varPhi }(\xi (x,\cdot ))\not \subseteq {\mathbb {E}}_{\varPhi }(\xi (x^*,\cdot )). $$

$$\square $$

## Main Results

In this section, we present the main theoretical findings of the study, which focus on key aspects of convexity and generalized convexity, optimality conditions, and sensitivity analysis. These results form the foundation for understanding the structure of the notion of a $\varPhi$-robust optimization problem and provide powerful tools for analyzing its solutions.

### Convexity Results

The following two results explore convexity and generalized convexity properties of the maps ${\mathbb {E}}_{\varPhi ^p}(\xi (x,\cdot ))$ and ${\mathbb {E}}_{\varPhi }(\xi (x,\cdot ))$ with respect to $p\in {{\mathbb {S}}}^*$ and $x\in X$, respectively.

#### Theorem 6.1

Let us define

$$g(x,p)={\mathbb {E}}_{\varPhi ^p}(\xi (x,\cdot )=spt({\mathbb {E}}_\varPhi (\xi (x,\cdot ),p))=\int _\varOmega \xi (x,\omega ) d\varPhi ^p(\omega ). $$

Then *g* is convex with respect to *p*.

#### Proof

The proof follows from the support of a set is a convex function with respect to *p*. In fact, we have

14 $$\begin{aligned} g(x,t p_1 + (1-t)p_2 = spt({\mathbb {E}}_\varPhi (\xi (x,\cdot ),t p_1 + (1-t) p_2)) = \end{aligned}$$

$$ \sup _{y\in {\mathbb {E}}_\varPhi (\xi (x,\cdot )} y\cdot (t p_1 + (1-t) p_2) \le t g(x, p_1) + (1-t) g(x,p_2) $$

$$\square $$

#### Theorem 6.2

If $\xi (x, \omega )$ is convex w.r.t. $x\in X$ and *X* is a convex set, then ${\mathbb {E}}_{\varPhi }(\xi (x,\cdot ))$ is a convex map. Furthermore, if $\xi$ is either quasi-convex or convex by arcs with respect to *x* then the same is true for ${\mathbb {E}}_\varPhi (\xi (x,\cdot ))$.

#### Proof

Assume $\xi$ is convex w.r.t. $x \in X$. Then for every $p \in {{\mathbb {S}}}^*$ it holds

15 $$\begin{aligned} {\mathbb {E}}_{\varPhi ^p} (\xi (tx+(1-t)y, \cdot ))\le t {\mathbb {E}}_{\varPhi ^p}(\xi (x, \cdot ))+(1-t){\mathbb {E}}_{\varPhi ^p}(\xi (y, \cdot )) \end{aligned}$$

for every $t \in [0,1]$, $x, y \in X$. This is exactly the same as

$$ spt({\mathbb {E}}_{\varPhi } (\xi (tx+(1-t)y, \cdot ),p))\le spt(t {\mathbb {E}}_{\varPhi }(\xi (x, \cdot ))+(1-t){\mathbb {E}}_{\varPhi }(\xi (y, \cdot )),p) $$

for any $p\in {{\mathbb {S}}}^*$. This implies that

$$ {\mathbb {E}}_{\varPhi } (\xi (tx+(1-t)y, \cdot ))\subseteq t {\mathbb {E}}_{\varPhi }(\xi (x, \cdot ))+(1-t){\mathbb {E}}_{\varPhi }(\xi (y, \cdot )), $$

and thus the function $x \mapsto {\mathbb {E}}_{\varPhi }(\chi (x,\cdot ))$ is a convex set-valued map. In a similar manner we can prove the thesis under the quasi-convexity and the convexity by arcs assumptions. $\square$

### Optimality Results

#### Theorem 6.3

We have that $x^*\in X$ is an ideal global minimizer for the problem

16 $$\begin{aligned} \textrm{min}_{x \in X}\ {\mathbb {E}}_{\varPhi }(\xi (x,\cdot )) \end{aligned}$$

if and only if $x^*$ is global minimizer to the problem

$$\begin{aligned} \textrm{min}_{x \in X}\ {\mathbb {E}}_{\varPhi ^p}(\xi (x,\cdot )) \end{aligned}$$

for all $$p\in {{\mathbb {S}}}^*$$.

#### Proof

Since $x^*$ is an ideal global minimizer for (16), it holds that:

17 $$\begin{aligned} {\mathbb {E}}_{\varPhi }(\xi (x^*,\cdot ))\subseteq {\mathbb {E}}_{\varPhi }(\xi (x,\cdot )) =\int _\varOmega \xi (x,\omega )d\varPhi (\omega ). \end{aligned}$$

This implies that:

18 $$\begin{aligned}  &   {\mathbb {E}}_{\varPhi ^p}(\xi (x^*,\cdot )) = \int _\varOmega \xi (x^*,\omega ) d\varPhi ^p = spt\left( \int _\varOmega \xi (x^*,\omega )d\varPhi (\omega ),p\right) \le \end{aligned}$$

19 $$\begin{aligned}  &   spt\left( \int _\varOmega \xi (x,\omega )d\varPhi (\omega ),p\right) = {\mathbb {E}}_{\varPhi ^p}(\xi (x,\cdot )) \end{aligned}$$

for any $x\in X$. This proves that $x^*$ is a minimizer of ${\mathbb {E}}_{\varPhi ^p}(\xi (x,\cdot ))$ for any $p\in {{\mathbb {S}}}^*$.

Conversely, since $x^*\in X$ is a solution to the scalarized problem for any $p\in {{\mathbb {S}}}^*$, then we have:

20 $$\begin{aligned} spt\left( {\mathbb {E}}_{\varPhi }(\xi (x^*,\cdot )),p\right) ={\mathbb {E}}_{\varPhi ^p}(\xi (x^*,\cdot ))\le {\mathbb {E}}_{\varPhi ^p}(\xi (x,\cdot ))=spt\left( {\mathbb {E}}_{\varPhi }(\xi (x,\cdot )),p\right) \end{aligned}$$

for any $x\in X$. Now the thesis follows by using a classical separation argument. $\square$

#### Theorem 6.4

For $x^* \in X$ consider the function

$$ g_{x^*}(x)=\sup _{p\in {{\mathbb {S}}}^*}\left( {\mathbb {E}}_{\varPhi ^p}(\xi (x, \cdot ))- {\mathbb {E}}_{\varPhi ^p}(\xi (x^*, \cdot )\right) . $$

Then if $x^*$ is a global strong minimizer for problem (9) then $x^*$ is also a solution to

21 $$\begin{aligned} \min _{x \in X}g_{x^*}(x). \end{aligned}$$

Conversely, if $x^*$ is a strict minimizer for (21), then $x^*$ is a strong global minimizer for (9). If $x^*$ is a minimizer for problem (21), then it is a minimizer for problem (9).

#### Proof

Assume $x^*\in X$ is a strong minimizer for problem (9). Hence, for every $x \in X$ we have

$$ {\mathbb {E}}_{\varPhi }(\xi (x, \cdot ))\not \subseteq {\mathbb {E}}_{\varPhi }(\xi (x^*, \cdot )). $$

This implies that for every $x \in X$ there exists $p \in {{\mathbb {S}}}^*$ such that $spt (p, {\mathbb {E}}_{\varPhi }(\xi (x, \cdot ))) \ge spt (p, {\mathbb {E}}_{\varPhi }(\xi (x^*, \cdot )))$ or equivalently ${\mathbb {E}}_{\varPhi ^p}(\xi (x, \cdot )) \ge {\mathbb {E}}_{\varPhi ^p}(\xi (x^*, \cdot ))$. This entails

$$ \sup _{p \in {{\mathbb {S}}}^*} \left( {\mathbb {E}}_{\varPhi ^p}(\xi (x, \cdot ))- {\mathbb {E}}_{\varPhi ^p}(\xi (x^*, \cdot ))\right) \ge 0 $$

which means $x^*$ is minimal for $g_{x^*}(x)$, since $g_{x^*}(x^*)=0$.

Assume now that $x^* \in X$ is a strict minimum for $g_{x^*}(x)$, that is that $g_{x^*}(x) > g_{x^*}(x^*) = 0$ for all $x \ne x^*$. Then for any $x \ne x^*$ we have that there is some $p \in {{\mathbb {S}}}^*$ with

$$ spt(p,{\mathbb {E}}_{\varPhi }(\xi (x^*,\cdot ))) < spt(p,{\mathbb {E}}_{\varPhi }(\xi (x,\cdot ))) $$

which means that ${\mathbb {E}}_{\varPhi }(\xi (x,\cdot )) \not \subseteq {\mathbb {E}}_{\varPhi }(\xi (x^*,\cdot ))$ and so $x^*$ is a strong minimizer as claimed. Assume $x^* \in X$ is a minimum of $g_{x^*}(x)$. For every *n* we have the existence of $p\in S^*$ such that

$$ spt(p,{\mathbb {E}}_{\varPhi }(\xi (x^*,\cdot ))) \le spt(p,{\mathbb {E}}_{\varPhi }(\xi (x,\cdot ))) $$

this means that we cannot have $spt(p,{\mathbb {E}}_{\varPhi }(\xi (x^*,\cdot ))) > spt(p,{\mathbb {E}}_{\varPhi }(\xi (x,\cdot )))$ for every $p \in S^*$, which is equivalent to ${\mathbb {E}}_{\varPhi }(\xi (x,\cdot ))\not \subset \textrm{int}\, {\mathbb {E}}_{\varPhi }(\xi (x^*,\cdot ))$ for every $x \in X$ and the proof is complete. $\square$

#### Corollary 6.1

If for some $p^* \in {{\mathbb {S}}}^*$, $x^* \in X$ is a strict minimizer for

$$ \min _{x \in X}spt(p^*, {\mathbb {E}}_{\varPhi }(\xi (x, \cdot )) $$

i.e.

$$ {\mathbb {E}}_{\varPhi ^{p^*}}(\xi (x^*, \cdot )) < {\mathbb {E}}_{\varPhi ^{p^*}}(\xi (x,\cdot )) \ \text{ for } \text{ all } x \ne x^*, $$

then $x^*$ is a strong minimizer for problem (9).

#### Proof

This follows directly from the argument given in the theorem. $\square$

### Stability Results

The following are stability results with respect to perturbations of the underlying set-valued probability, the objective function and the feasible set, respectively. In what follows, even if it is not explicitly mentioned, we assume that the function $\xi (x,\omega )$ is Lipschitz with respect to both *x* and $\omega$ and we assume $\varOmega$ is also a metric space. This hypothesis guarantees the convergence of the Monge-Kantorovich distance.

#### Theorem 6.5

Let $\xi :X \times \varOmega \rightarrow {\mathbb {R}}$ be Lipschitz on the product space $X \times \varOmega$, let $\varPhi _n$ be a sequence of set-valued probabilities that are converging to $\varPhi$ in the extended Monge-Kantorovich metric, and let $x_n\in X$ be a sequence converging to $x^*\in X$. If $x_n$ is a global ideal minimizer for the problem

22 $$\begin{aligned} \textrm{min}_{x \in X}\ {\mathbb {E}}_{\varPhi _n}(\xi (x,\cdot )) \end{aligned}$$

then $x^*\in X$ is a global ideal minimizer for the problem

23 $$\begin{aligned} \textrm{min}_{x \in X}\ {\mathbb {E}}_{\varPhi }(\xi (x,\cdot )). \end{aligned}$$

#### Proof

We have

$$ \sup _{p \in {{\mathbb {S}}}^*} \left| \int _{\varOmega }\xi (x, \omega )d\varPhi ^p_n(\omega )-\int _{\varOmega }\xi (x, \omega )d\varPhi ^p(\omega )\right| \le \sup _{p\in {{\mathbb {S}}}^*} d_{{\textbf{M}}}(\varPhi ^p_n, \varPhi ^p)= d_{{\textbf{M}}}(\varPhi _n, \varPhi ). $$

Hence

$$ {\mathbb {E}}_{\varPhi ^p_n}(\xi (x, \cdot )) \rightarrow {\mathbb {E}}_{\varPhi ^p}(\xi (x, \cdot )), $$

for every $p \in {{\mathbb {S}}}^*$. Similarly,

$$\begin{aligned}  &   \sup _{p \in {{\mathbb {S}}}^*} \left| \int _{\varOmega }\xi (x_n, \omega )d\varPhi ^p_n(\omega )-\int _{\varOmega }\xi (x, \omega )d\varPhi ^p(\omega )\right| \\  &   \le \sup _{p \in {{\mathbb {S}}}^*} \left| \int _{\varOmega }(\xi (x_n, \omega )-\xi (x, \omega ))d\varPhi ^p_n(\omega )-\int _{\varOmega }\xi (x, \omega )d\varPhi ^p(\omega )+\int _{\varOmega }\xi (x, \omega )\varPhi ^p_n(\omega )\right| \\  &   \le \sup _{p \in {{\mathbb {S}}}^*} \left| \int _{\varOmega }(\xi (x_n, \omega )-\xi (x, \omega ))d\varPhi ^p_n(\omega )\right| + \sup _{p \in {{\mathbb {S}}}^*} \left| \int _{\varOmega }\xi (x, \omega )d\varPhi ^p(\omega )-\int _{\varOmega }\xi (x, \omega )\varPhi ^p_n(\omega )\right| \\  &   \le \sup _{\omega \in \varOmega }\left| \xi (x_n, \omega )-\xi (x, \omega )\right| +d_{{\textbf{M}}}(\varPhi _n, \varPhi ) \le K d(x_n,x) + d_M(\varPhi _n,\varPhi ), \end{aligned}$$

where *K* is the Lipschitz constant for $\xi$. Hence

$$ {\mathbb {E}}_{\varPhi _n}(\xi (x_n, \cdot ))\rightarrow {\mathbb {E}}_{\varPhi }(\xi (x, \cdot )) $$

and the proof now follows immediately. $\square$

#### Theorem 6.6

Let *X* be a compact set, let $\xi _n \rightarrow \xi$ in the pointwise convergence and let $x_n\in X$ be a sequence converging to $x^*\in X$. If $x_n$ is an ideal minimizer of the problem

24 $$\begin{aligned} \textrm{min}_{x \in X}\ {\mathbb {E}}_{\varPhi }(\xi _n(x,\cdot )) \end{aligned}$$

for every *n*, then $x^*\in X$ is an ideal minimizer of the problem

25 $$\begin{aligned} \textrm{min}_{x \in X}\ {\mathbb {E}}_{\varPhi }(\xi (x,\cdot )). \end{aligned}$$

#### Proof

Since $x_n$ is a solution of problem (24), we have

$$ {\mathbb {E}}_{\varPhi ^p}(\xi (x_n, \cdot )) \le {\mathbb {E}}_{\varPhi ^p}(\xi _n(x, \cdot )) $$

for every $x \in X$ and $p \in {{\mathbb {S}}}^*$, i.e.

$$ \int _{\varOmega }\xi _n(x_n, \omega )d\varPhi ^p(\omega )\le \int _{\varOmega }\xi _n(x, \omega )d\varPhi ^p(\omega ). $$

Since $\xi _n$ converges to $\xi$ pointwise, passing to the limit we obtain $\xi _n(x_n, \omega ) \rightarrow \xi (x^*, \omega )$. We have

$$\begin{aligned}  &   \left| {\mathbb {E}}_{\varPhi ^p}(\xi _n(x_n, \cdot )-{\mathbb {E}}_{\varPhi ^p}(\xi (x^*, \cdot )\right| = \left| \int _{\varOmega }\xi _n(x_n, \omega )d\varPhi ^p(\omega )- \int _{\varOmega }\xi (x^*, \omega )d\varPhi ^p(\omega )\right| \\  &   \le \int _{\varOmega }\left| \xi _n(x_n, \omega )-\xi (x^*, \omega )\right| d\varPhi ^p(\omega ) \le \Vert \xi _n-\xi \Vert _{\infty }. \end{aligned}$$

Recalling that if *X* is compact continuous convergence implies uniform convergence, we get ${\mathbb {E}}_{\varPhi ^p}(\xi _n(x_n, \cdot ))\rightarrow {\mathbb {E}}_{\varPhi ^p}(\xi (x^*, \cdot ))$. Similarly ${\mathbb {E}}_{\varPhi ^p}(\xi _n(x, \cdot )) \rightarrow {\mathbb {E}}_{\varPhi ^p}(\xi (x, \cdot ))$ and we get

$$ {\mathbb {E}}_{\varPhi ^p}(\xi (x^*, \cdot )) \le {\mathbb {E}}_{\varPhi ^p}(\xi (x, \cdot )) $$

for every $p \in {{\mathbb {S}}}^*$ which completes the proof. $\square$

#### Theorem 6.7

Let *X* be a compact set, let $X_n$ be a sequence of sets $X_n\subseteq X_{n+1}\subseteq X$ with $\bigcup _{n\ge 0} X_n=X$. Suppose that $\xi$ is a continuous function with respect to $x\in X$ for any $\omega \in \varOmega$ fixed. Let $x_n\in X_n$ be a sequence converging to $x^*\in X$. If $x_n$ is an ideal minimizer for the problem

$$ \min _{x\in X_n} {\mathbb {E}}_\varPhi (\xi (x,\cdot )) $$

then $x^*\in X$ is an ideal minimizer for the problem

$$ \min _{x\in X} {\mathbb {E}}_\varPhi (\xi (x,\cdot )). $$

#### Proof

For any $x\in X$ there exists $n_0$ such that for any $n\ge n_0$ we have that

$$ {\mathbb {E}}_\varPhi (\xi (x_n,\cdot ))\subseteq {\mathbb {E}}_\varPhi (\xi (x,\cdot )). $$

This implies that for any direction $p\in {{\mathbb {S}}}^*$ we have that

$$ {\mathbb {E}}_{\varPhi ^p}(\xi (x_n,\cdot ))\le {\mathbb {E}}_{\varPhi ^p}(\xi (x,\cdot )). $$

By taking the limit when $n\rightarrow +\infty$ and using the Lebesgue dominated convergence theorem, we deduce that

$$ {\mathbb {E}}_{\varPhi ^p}(\xi (x^*,\cdot ))\le {\mathbb {E}}_{\varPhi ^p}(\xi (x,\cdot )), $$

which proves the thesis. $\square$

Stability results for minimizers of problem (9) can be given similarly. As an example we consider the following result.

#### Theorem 6.8

Let *X* be a compact set and let $\xi _n\rightarrow \xi$ uniformly and let $x_n \in X$ be a sequence converging to $x^* \in X$. If $x_n\in X$ is a minimizer of the problem

26 $$\begin{aligned} \textrm{min}_{x \in X}\ {\mathbb {E}}_{\varPhi }(\xi _n(x,\cdot )) \end{aligned}$$

for every *n*, then $x^*\in X$ is a minimizer of the problem

27 $$\begin{aligned} \textrm{min}_{x \in X}\ {\mathbb {E}}_{\varPhi }(\xi (x,\cdot )). \end{aligned}$$

#### Proof

Fix $x \in X$. Since $x_n$ is a solution of problem (26), by Theorem 6.4 we have

28 $$\begin{aligned} \sup _{p \in {{\mathbb {S}}}^*}({\mathbb {E}}_{\varPhi ^p}(\xi _n(x, \cdot )-{\mathbb {E}}_{\varPhi ^p}(\xi _n(x_n, \cdot ))\ge 0. \end{aligned}$$

This entails that for every *n* there exists $p_n \in S^*$ such that

29 $$\begin{aligned} {\mathbb {E}}_{\varPhi ^{p_n}}(\xi _n(x, \cdot )-{\mathbb {E}}_{\varPhi ^{p_n}}(\xi _n(x_n, \cdot )\ge 0. \end{aligned}$$

Using compactness of ${{\mathbb {S}}}^*$ and passing to a subsequence if necessary we know that

$$ {\mathbb {E}}_{\varPhi ^{p_n}}(\xi _n(x, \cdot )\rightarrow {\mathbb {E}}_{\varPhi ^{p_*}}(\xi (x, \cdot ) $$

for some $p^* \in {{\mathbb {S}}}^*$. Furthermore, from the uniform convergence of $\xi _n \rightarrow \xi$ we have

$$ {\mathbb {E}}_{\varPhi ^{p_n}}(\xi (x_n, \cdot )\rightarrow {\mathbb {E}}_{\varPhi ^{p_*}}(\xi (x^*, \cdot ). $$

This entails

30 $$\begin{aligned} {\mathbb {E}}_{\varPhi ^{p^*}}(\xi (x, \cdot )-{\mathbb {E}}_{\varPhi ^{p^*}}(\xi (x_n, \cdot )\ge 0 \end{aligned}$$

and hence

31 $$\begin{aligned} \sup _{p\in S^*}({\mathbb {E}}_{\varPhi ^{p}}(\xi (x, \cdot )-{\mathbb {E}}_{\varPhi ^{p}}(\xi (x_n, \cdot )\ge 0, \end{aligned}$$

which concludes the proof. $\square$

## $\varPhi$-Robustness in Finance: Two Applications

Robust optimization has become an indispensable framework for decision-making under uncertainty, particularly within the financial sector where unpredictability and risk are inherent. Unlike traditional optimization methods that rely on fixed, point-estimate probabilities, robust optimization employs set-valued probabilities, allowing for a more flexible and realistic modeling of uncertainty. By representing probabilities as sets rather than fixed values, this approach accommodates the ambiguity and variability often present in real-world scenarios, enabling decision-makers to account for a range of possible outcomes rather than relying on potentially inaccurate assumptions.

One key application of the notion of $\varPhi$-robustness is financial portfolio optimization. Investors face the complex challenge of allocating assets to maximize expected returns while minimizing risk, all within the context of highly volatile and unpredictable financial markets. Traditional portfolio optimization methods often fail to account for the uncertainty in estimating future market probabilities, which can render portfolios vulnerable to adverse outcomes. $\varPhi$-robust portfolio optimization, on the other hand, uses set-valued probabilities (more specifically, in our example, we use interval-valued probabilities) to proactively safeguard against worst-case scenarios. This ensures that portfolios are resilient across a wide spectrum of potential market conditions, providing investors with a more reliable strategy to protect their assets from significant losses. By enhancing the robustness of investment strategies, this method aligns with the goals of risk-averse investors who prioritize long-term stability over speculative gains.

Another interesting application of the notion of $\varPhi$-robustness is robust risk minimization, which focuses on mitigating the exposure to financial risks under uncertain conditions. In the context of risk management for financial institutions, robust optimization can be used to model credit risks, market risks, or operational risks with set-valued probability. This approach enables financial institutions to develop strategies that minimize downside risks, even when the precise probabilities of adverse events-such as default rates or market crashes-are unknown. By adopting robust risk minimization techniques, decision-makers can effectively balance potential losses against acceptable levels of risk, ensuring that financial systems remain stable even in turbulent environments.

### A $\varPhi$-Robust Formulation of Markowitz Portfolio Model

The Markowitz Portfolio Problem [20], developed by Harry Markowitz in the 1950s, is a foundational concept in modern portfolio theory. It seeks to determine the optimal allocation of assets in a portfolio to maximize expected returns while minimizing risk. The core idea is to create a portfolio that achieves the best possible trade-off between risk and return, based on the historical performance of various assets.

The portfolio expected return ${\mathbb {E}}(R)$ is calculated as ${\mathbb {E}}(R) = \sum _{i=1}^{n} w_i {\mathbb {E}}(R_i)$ where $w_i$ is the weight of asset $i$ in the portfolio, ${\mathbb {E}}(R_i)$ is the expected return of asset $i$, and $n$ is the number of assets in the portfolio.

Risk, often measured by the variance of the portfolio $\sigma ^2$, is given by $\sigma ^2 = \sum _{i=1}^{n} \sum _{j=1}^{n} w_i w_j \sigma _{ij}$ where $\sigma _{ij}$ is the covariance between the returns of assets $i$ and $j$. One of the key outcomes of this theory is the efficient frontier, which represents the set of optimal portfolios that offer the highest expected return for a given level of risk. Diversification is a crucial strategy in this context, as it involves investing in a variety of assets to reduce risk. By spreading investments across different assets, investors can mitigate the impact of poor performance in any single investment.

The *deterministic equivalent problem* is a formulation used in decision-making under uncertainty. In the context of the Markowitz Portfolio Problem, it involves converting a stochastic (random) problem into a deterministic one. Formally, the deterministic equivalent of a stochastic optimization problem is defined as:

$$ \text {Maximize } {\mathbb {E}}(R) \text { subject to } \sigma ^2 \le \sigma ^2_{\text {target}}, $$

where $\sigma ^2_{\text {target}}$ is the maximum allowable risk. This approach allows investors to create a simplified model that focuses on expected returns and risks without accounting for the inherent uncertainty in the market. The deterministic equivalent facilitates easier analysis and optimization of the portfolio, enabling the identification of the optimal asset allocation.

Both the expected values and the covariances are computed with respect to an underlying probability measure $\mu$ that reflects the distribution of possible returns and volatility. Let $\varPhi _\mu$ be the set-valued probability induced by $\mu$, that is $\varPhi _\mu (A)=[0,\mu (A)]$ and let $\varPhi$ be a set-valued probability such that $\varPhi _\mu (A)\subseteq \varPhi (A)$, for any measurable set *A*. The robust version of the Markowitz model with respect to $\varPhi$ is then formulated as

$$ \text {Maximize } {\mathbb {E}}_\varPhi (R) \text { subject to } \sigma ^2 \le \sigma ^2_{\text {target}}. $$

In order to illustrate how this approach works in a practical context, let us consider the following case study which focuses on optimizing a portfolio comprising ten prominent tech companies, emphasizing expected returns in both optimistic and pessimistic economic scenarios. The selected assets for this portfolio include: Apple Inc. (AAPL), Microsoft Corp. (MSFT), Alphabet Inc. (GOOGL), Amazon.com Inc. (AMZN), Meta Platforms Inc. (META), NVIDIA Corp. (NVDA), Tesla Inc. (TSLA), Adobe Inc. (ADBE), Salesforce.com Inc. (CRM), Netflix Inc. (NFLX). The expected returns for the assets were estimated based on historical performance and market analysis.

Let us consider two probability measures, one describing the optimistic scenario and denoted by $\mu _{opt}$ and one the pessimistic scenario $\mu _{pes}$. Associated with these two probabilities, one can build a set-valued (interval-valued) probability measure as explained in the first part of this paper, namely: $\varPhi (A) = [-\mu _{pes}(A),\mu _{opt}(A)]$. The expected returns corresponding to the optimistic and pessimistic scenarios are:

*Optimistic Scenario*

$$ R_{optimistic} = [0.18, 0.15, 0.14, 0.16, 0.12, 0.20, 0.17, 0.13, 0.11, 0.10] $$

*Pessimistic Scenario*

$$\begin{aligned} R_{pessimistic} = [0.08, 0.06, 0.05, 0.07, 0.04, 0.09, 0.07, 0.05, 0.03, 0.02] \end{aligned}$$

while the covariance matrix reflecting the relationships between the assets is given by:

$$\begin{aligned} \varSigma = \begin{bmatrix} 0.005 &  0.003 &  0.002 &  0.002 &  0.002 &  0.003 &  0.002 &  0.002 &  0.002 &  0.001 \\ 0.003 &  0.006 &  0.002 &  0.002 &  0.002 &  0.002 &  0.002 &  0.002 &  0.001 &  0.001 \\ 0.002 &  0.002 &  0.007 &  0.002 &  0.001 &  0.002 &  0.001 &  0.001 &  0.002 &  0.001 \\ 0.002 &  0.002 &  0.002 &  0.006 &  0.002 &  0.002 &  0.002 &  0.002 &  0.001 &  0.001 \\ 0.002 &  0.002 &  0.001 &  0.002 &  0.005 &  0.001 &  0.001 &  0.001 &  0.001 &  0.001 \\ 0.003 &  0.002 &  0.002 &  0.002 &  0.001 &  0.006 &  0.002 &  0.001 &  0.002 &  0.001 \\ 0.002 &  0.002 &  0.001 &  0.002 &  0.001 &  0.002 &  0.005 &  0.002 &  0.002 &  0.001 \\ 0.002 &  0.002 &  0.001 &  0.002 &  0.001 &  0.001 &  0.002 &  0.005 &  0.001 &  0.001 \\ 0.002 &  0.001 &  0.002 &  0.001 &  0.001 &  0.002 &  0.002 &  0.001 &  0.003 &  0.001 \\ 0.001 &  0.001 &  0.001 &  0.001 &  0.001 &  0.001 &  0.001 &  0.001 &  0.001 &  0.002 \end{bmatrix} \end{aligned}$$

The constraints include:

- The sum of weights $w_i$ must equal 1.
- Portfolio volatility $\sigma ^2 = w^T \varSigma w$ must be less than or equal to $\sigma ^2_{\text {target}}=0.003$.
- Each weight $w_i$ must be nonnegative.

The robust formulation of the portfolio optimization problem reads as:

32 $$\begin{aligned} \text {Maximize } {\mathbb {E}}_\varPhi (R) \end{aligned}$$

subject to

33 $$\begin{aligned} \sum _{i=1}^{10} w_i = 1, \ \ w_i\ge 0, \ \ w^T \varSigma w \le \sigma ^2_{\text {target}}, \end{aligned}$$

where

$$\begin{aligned}  &   {\mathbb {E}}_\varPhi (R)(w_1,...w_{10}) = w_1 [-0.08,0.18] + w_2 [-0.06,0.15] + w_3 [-0.05,0.14] + +\\  &   w_4 [-0.07,0.16] + w_5 [-0.04,0.12] + w_6 [-0.09,0.20] + w_7 [-0.07,0.17] + \\  &   w_8 [-0.05,0.14] + w_9 [-0.03,0.11] + w_{10} [-0.02,0.10]. \end{aligned}$$

The goal of the robust formulation was to minimize the expected return in Eq. (32) while keeping the portfolio volatility below a specified threshold. The problem is set-valued and an optimal solution consists of a vector of weights $w_i^*$, $i=1..10$, such that ${\mathbb {E}}_\varPhi (R)(w_1,...w_{10})\subseteq {\mathbb {E}}_\varPhi (R)(w_1^*,...w_{10}^*)$. The optimal solution corresponds, therefore, to the largest interval that includes all other possible outcomes. Therefore, this problem can be stated as a bicriteria problem which reads as:

34 $$\begin{aligned} \text {Maximize} \ {\mathbb {E}}_\varPhi (R)(w_1,...w_{10}) = ({\mathbb {E}}_{\mu _{pes}}(R)(w_1,...w_{10}),{\mathbb {E}}_{\mu _{opt}}(R)(w_1,...w_{10})), \nonumber \\ \end{aligned}$$

where

$$\begin{aligned}  &   {\mathbb {E}}_{\mu _{pes}}(R)(w_1,...w_{10}) = 0.08 w_1 + 0.06 w_2 + 0.05 w_3 + 0.07 w_4 + 0.04 w_5+ \\  &   0.09 w_6 + 0.07 w_7 + 0.05 w_8 + 0.03 w_9 + 0.02 w_{10} \end{aligned}$$

and

$$\begin{aligned}  &   {\mathbb {E}}_{\mu _{opt}}(R)(w_1,...w_{10}) = 0.18 w_1 + 0.15 w_2 + 0.14 w_3 + 0.16 w_4 + 0.12 w_5+ \\  &   0.20 w_6 + 0.17 w_7 + 0.14 w_8 + 0.11 w_9 + 0.10 w_{10}. \end{aligned}$$

In order to determine a compromise solution, let us scalarize the problem with some trade-off parameter $\lambda \in [0,1]$, which reads as:

35 $$\begin{aligned} \text {Maximize\,\,} {\mathbb {E}}_\varPhi (R)(w_1,...w_{10})= &   \lambda {\mathbb {E}}_{\mu _{pes}}(R)(w_1,...w_{10}) \nonumber \\  &   + (1-\lambda ) {\mathbb {E}}_{\mu _{opt}}(R)(w_1,...w_{10}). \end{aligned}$$

The optimal solution of Eq. (35) which balances the optimistic and the pessimistic scenarios with $\lambda =0.5$, is given by

$$ \textbf{w} = [0.235 \ 0 \ 0.029 \ 0.136 \ 0 \ 0.335 \ 0.243 \ 0.022 \ 0 \ 0] $$

with expected portfolio return of 0.128. The resulting portfolio allocations for both scenarios ($\lambda =0$ and $\lambda =1$) are, instead, as follows:

*Optimistic Portfolio Weights* ($\lambda = 0$):

$$ {\textbf{w}_{\textbf{opt}}}= [0.222 \ 0 \ 0.039 \ 0.120 \ 0.002 \ 0.343 \ 0.259 \ 0.015 \ 0 \ 0] $$

and the expected portfolio return is 0.179.

*Pessimistic Portfolio Weights* ($\lambda =1$):

$$ {\textbf{w}_{\textbf{pes}}}= [0.249 \ 0 \ 0.005 \ 0.164 \ 0 \ 0.327 \ 0.216 \ 0.039 \ 0 \ 0] $$

and the expected portfolio return is equal to 0.078.

This case study successfully demonstrates the application of robust portfolio optimization techniques. By carefully selecting assets and employing robust mathematical models, investors can strategically manage risks while seeking optimal returns.

### A $\varPhi$-Robust Formulation of the Classical Risk Minimization Problem

In this subsection, we introduce a $\varPhi$-robust variant of the classical risk minimization problem. Let us consider a probability space $(\varOmega , \mathcal {F}, \mu )$, where $\varOmega$ is the sample space, $\mathcal {F}$ is the $\sigma$-algebra of events, and $\mathcal {A}$ is a set of potential probability measures. For a random variable $X : \varOmega \rightarrow \mathbb {R}$, a coherent risk measure ( [3, 15]) $\rho (X)$ is defined as:

36 $$\begin{aligned} \rho (X) = \sup _{\mu \in \mathcal {A}} \mathbb {E}_{\mu }(-X), \end{aligned}$$

where $\mathbb {E}_{\mu }$ denotes the expectation with respect to the probability measure $\mu$. Suppose now we have *d* sets of coherent risk measures denoted by $\rho _i$. We can build a vector-valued coherent risk measure by considering the following vector-valued functional

37 $$\begin{aligned} \rho (X) = \left( \rho _1(X),\ldots ,\rho _d(X)\right) = \left( \sup _{\mu \in \mathcal {A}_1} \mathbb {E}_{\mu }(-X),\ldots , \sup _{\mu \in \mathcal {A}_d} \mathbb {E}_{\mu }(-X)\right) . \end{aligned}$$

Now, assume that the random variable *X* can be expressed as a linear combination of *n* random assets, i.e., $X = \sum _{i=1}^n w_i X_i$ where $X_i$ represents the return of the *i*-th asset, and $w_i$ is the budget allocation assigned to that asset. The classical risk minimization problem then takes the following form:

38 $$\begin{aligned} \min _{w_1, \dots , w_n} \left( \rho _{1}\left( \sum _{i=1}^n w_i X_i\right) ,\ldots ,\rho _d\left( \sum _{i=1}^n w_i X_i\right) \right) , \end{aligned}$$

subject to the following constraints:

39 $$\begin{aligned} \sum _{i=1}^n w_i = 1, \quad 0 \le w_i \le 1, \quad \forall i \in \{1, \dots , n\}. \end{aligned}$$

Here, the constraint $\sum _{i=1}^n w_i = 1$ ensures that the budget is fully allocated, while $0 \le w_i \le 1$ reflects the practical requirement that individual allocations cannot be negative or exceed the total budget.

We now extend this classical framework by incorporating $\varPhi$-robustness. Given a set-valued probability $\varPhi$ taking values in ${\mathbb {R}}^d$, a $\varPhi$ coherent risk measure is defined as $\rho _\varPhi (X) = {\mathbb {E}}_\varPhi (-X)$. As in the previous part of this paper, the introduction of the set-valued measure encompasses the sets of probability distributions $\mathcal {A}_i$. With respect to $\rho _\varPhi$, the $\varPhi$-robust formulation of the risk minimization problem reads as

40 $$\begin{aligned} \min _{w_1, \dots , w_n} \rho _\varPhi \left( \sum _{i=1}^n w_i X_i\right) = \min _{w_1, \dots , w_n} \mathbb {E}_\varPhi \left( -\sum _{i=1}^n w_i X_i\right) , \end{aligned}$$

subject to the previous budget and sign constraints. The following result presents some properties of the risk measure $\rho _\varPhi$ which are analogous to well-known properties of single-valued coherent risk measures.

#### Theorem 7.1

Given two positive random variables *X* and *Y*, a constant *c*, and a positive real number *t*, the risk measure $\rho _\varPhi$ satisfies the following properties:

1. $\rho _\varPhi (c) = c {\mathbb {B}}$ where ${\mathbb {B}}$ is the unit ball in ${\mathbb {R}}^d$,
2. $$\rho _\varPhi (X+Y) = \rho _\varPhi (X) + \rho _\varPhi (Y)$$,
3. $$\rho _\varPhi (tX) = t \rho _\varPhi (X)$$,
4. $$\rho _\varPhi (tX+(1-t)Y) = t\rho _\varPhi (X) + (1-t)\rho _\varPhi (Y)$$ for $$0 \le t \le 1$$,
5. If $X\le Y\le c$, where *c* is positive constant, then $\rho _\varPhi (c-Y)\subseteq \rho _\varPhi (c-X)$.

#### Proof

1. We have that $\rho _\varPhi (c) = {\mathbb {E}}_\varPhi (-c) d\varPhi = -c \varPhi (\varOmega ) = - c {\mathbb {B}}= c {\mathbb {B}}.$
2. Computing, we have:
  $$\begin{aligned} \rho _\varPhi (X+Y)&= {\mathbb {E}}_{\varPhi } (-(X+Y)) = \int _\varOmega -X-Y d\varPhi \\&= \int _\varOmega -X d\varPhi + \int _\varOmega -Y d\varPhi = \rho _\varPhi (X) + \rho _\varPhi (Y). \end{aligned}$$
3. We have $$\rho _\varPhi (t X) = {\mathbb {E}}_\varPhi (-tX) = t {\mathbb {E}}_\varPhi (-X) = t \rho _\varPhi (X)$$.
4. This one follows from the first two properties.
5. If $X\le Y\le c$ where *c* is a positive constant, then $0\le c-Y \le c-X$ and for any $p\in S^*$, we have
  $$\begin{aligned} spt(p,\rho _{\varPhi }(Y-c))&= spt(p,{\mathbb {E}}_\varPhi (c-Y)) = \int _\varOmega c-Y d\varPhi ^p(\omega )\\&\le \int _\varOmega c-X d\varPhi ^p(\omega ) = spt(p,\rho _\varPhi (c-X)) \end{aligned}$$
  and this implies that $\rho _{\varPhi }(c-Y)\subseteq \rho _{\varPhi }(c-X)$.

$$\square $$

Let us notice that from a practical implementation perspective, the set-valued optimization model

41 $$\begin{aligned} \min _{w_1, \dots , w_n} \mathbb {E}_\varPhi \left( -\sum _{i=1}^n w_i X_i\right) , \end{aligned}$$

can be scalarized by means of support functions and the reduced to a infinite family of parametrized scalar optimization models taking the form:

42 $$\begin{aligned} \min _{w_1, \dots , w_n} spt\left( \mathbb {E}_\varPhi \left( -\sum _{i=1}^n w_i X_i\right) ,p\right) , \end{aligned}$$

for $$p\in {{\mathbb {S}}}^*$$.

## Conclusions

The introduction of generalized robustness through set-valued probabilities provides a more flexible and sophisticated approach to modeling uncertainty, enabling more effective decision-making in complex environments. This unified framework enhances analytical rigor while simultaneously bolstering the resilience of solutions against the challenges introduced by ambiguity in probability measures. In this paper, we established optimality results based on the concept of $\varPhi$-robustness and demonstrated stability results that accommodate perturbations in the set-valued measure, the function $\xi$, and the feasible set. These theoretical contributions underline the robustness and adaptability of the proposed framework. The two financial applications discussed in the final section of the paper underscore the transformative potential of the notion of $\varPhi$ robustness. By leveraging set-valued probabilities, this approach strengthens the stability and resilience of financial strategies. It equips investors and institutions with practical tools to address the inherent uncertainties of financial markets, fostering decision-making that is both forward-looking and grounded in sound risk management principles.
